# Incidence of Bacteriocins Produced by Food-Related Lactic Acid Bacteria Active towards Oral Pathogens

**DOI:** 10.3390/ijms14034640

**Published:** 2013-02-26

**Authors:** Georgia Zoumpopoulou, Eudoxie Pepelassi, William Papaioannou, Marina Georgalaki, Petros A. Maragkoudakis, Petros A. Tarantilis, Moschos Polissiou, Effie Tsakalidou, Konstantinos Papadimitriou

**Affiliations:** 1 Laboratory of Dairy Research, Department of Food Science and Technology, Agricultural University of Athens, Iera Odos 75, Athens 11855, Greece; E-Mails: gz@aua.gr (G.Z.); mgeor@aua.gr (M.G.); petrosm@aua.gr (P.A.M.); et@aua.gr (E.T.); 2 Department of Periodontology, School of Dentistry, National and Kapodistrian University of Athens, 2 Thivon Str., Athens 11527, Greece; E-Mail: epepela@dent.uoa.gr; 3 Department of Preventive and Community Dentistry, School of Dentistry, National and Kapodistrian University of Athens, 2 Thivon Str., Athens 11527, Greece; E-Mail: vpapaio@dent.uoa.gr; 4 Laboratory of Chemistry, Department of Science, Agricultural University of Athens, Iera Odos 75, Athens 11855, Greece; E-Mails: ptara@aua.gr (P.A.T.); mopol@aua.gr (M.P.)

**Keywords:** lactic acid bacteria, food, oral, *Streptococcus*, bacteriocin, FT-IR

## Abstract

In the present study we investigated the incidence of bacteriocins produced by 236 lactic acid bacteria (LAB) food isolates against pathogenic or opportunistic pathogenic oral bacteria. This set of LAB contained several strains (≥17%) producing bacteriocins active against food-related bacteria. Interestingly only *Streptococcus macedonicus* ACA-DC 198 was able to inhibit the growth of *Streptococcus oralis*, *Streptococcus sanguinis* and *Streptococcus gordonii*, while *Lactobacillus fermentum* ACA-DC 179 and *Lactobacillus plantarun* ACA-DC 269 produced bacteriocins solely against *Streptococcus oralis*. Thus, the percentage of strains that were found to produce bacteriocins against oral bacteria was ~1.3%. The rarity of bacteriocins active against oral LAB pathogens produced by food-related LAB was unexpected given their close phylogenetic relationship. Nevertheless, when tested in inhibition assays, the potency of the bacteriocin(s) of *S. macedonicus* ACA-DC 198 against the three oral streptococci was high. Fourier-transform infrared spectroscopy combined with principal component analysis revealed that exposure of the target cells to the antimicrobial compounds caused major alterations of key cellular constituents. Our findings indicate that bacteriocins produced by food-related LAB against oral LAB may be rare, but deserve further investigation since, when discovered, they can be effective antimicrobials.

## 1. Introduction

Probiotics, defined as “live microorganisms which, when administered in adequate amounts, confer a health benefit on the host” [1], have become a major topic of lactic acid bacteria (LAB) research over the last two decades. Although a number of health-promoting effects arising from the use of probiotics have been demonstrated, so far the main field of research has been the gastrointestinal tract [2]. Probiotics have been poorly investigated from the oral health perspective. However, this is gradually changing [3].

Dental plaque is a biofilm that develops naturally on the teeth and is composed of about 700 bacteria species that take part in the complex ecosystem of the oral cavity [4]. Interactions among these bacterial species, from the early stages of colonization to the formation of mature supragingival and subgingival plaque, are the main etiological agents of oral infectious diseases, such as dental caries, gingivitis and periodontitis [5]. Dental caries is a multifactorial disease of bacterial origin that is characterized by acid demineralization of the tooth enamel as a result of the proliferation of streptococci from the mutans group [6]. Gingivitis and periodontitis are largely caused by specific Gram-negative bacterial infections. Such infections lead to the destruction of the connective tissue and the disruption of the underlying alveovar bone and periodontal ligament supporting the teeth [7]. Moreover, in recent years it has been suggested that oral bacteria are associated with many systemic diseases, such as pneumonia and cardiovascular disease [8,9].

Hence, the need for oral care in a systemic health regimen has been emphasized. Generally, dental plaques that have been deposited firmly as biofilms are removed mechanically while antibacterial mouthwashes are also effective in decreasing tooth surface plaque. In general, mouthwashes contain fluorides, alcohols, detergents or antibacterial substances. Ideal antibacterial substances must be effective against pathogenic or opportunistic pathogenic strains related to oral health, act rapidly, maintain activity at low concentrations, and have no side effects [10]. In this context, bacteriocins isolated from food related LAB could be used as alternative antimicrobials [11,12].

In the present study, the aim was to investigate the antimicrobial activity of LAB strains isolated from food products against oral pathogenic or opportunistic pathogenic bacteria. The inhibitory activity of food LAB strains was studied towards both Gram-negative and Gram-positive strains of bacteria associated with oral health. Moreover, the involvement of proteinaceous compound(s) to this activity was examined. Finally, Fourier-transform infrared (FT-IR) spectroscopy was employed in order to unravel the changes at the cellular level caused by these antimicrobial substances on target bacteria.

## 2. Results and Discussion

### 2.1. Screening LAB Food Isolates for the Production of Bacteriocins Active against Oral Pathogens

A total of 236 LAB strains of food origin (Table 1) were screened for antimicrobial activity against 10 target strains (3 Gram-negative and 7 Gram-positive).

Within the set of strains to be tested as bacteriocin-producers against oral bacteria, there were a number of strains that had been screened in our laboratory against food-related bacteria and were found to produce bacteriocins (Supplementary Table 1). From the total of 236 strains, approximately 140 had been previously screened and from those 41 were found positive based on the well diffusion assay (WDA, see Experimental Section). Thus, a conservative estimate of the percentage of bacteriocin-producing strains in the total of strains tested in the present study would be ≥17%. Different sets of target strains were used including common indicator strains employed by different laboratories in similar studies (e.g., *Lactococcus lactis* CNRZ 117, *Lactobacillus sakei* LMG 2313, *Listeria innocua* BL86/26, *etc.*), as well as in-house indicator strains (e.g., *Lactococcus lactis* ACA-DC 49, *Streptococcus thermophilus* ACA-DC 4, *Lactobacillus delbrueckii* subsp. *bulgaricus* ACA-DC 84, *etc.*). Even though for some of the commonly used indicator strains their food origin is established (e.g., for *Lactobacillus helveticus* ATCC 15009 and *L. lactis* CNRZ 117, which are isolated from dairy products), for others (e.g., *L. innocua* BL86/26) the isolation source is not available in the literature or in the databases of culture collections. Our in-house indicator strains were all food isolates of dairy origin.

In the current screening assay, in order to increase the possibility for bacteriocin production, all potential producers were grown in three different culture media, namely skim milk containing yeast extract, MRS and M17. The respective cell-free culture supernatants (CFCSs) were tested towards the target bacteria by the WDA. In case of positive results, the 10-fold concentrated CFCSs, obtained after ammonium sulfate precipitation, were also tested for antimicrobial activity against the sensitive bacteria by the WDA.

None of the LAB strains was able to inhibit any of the Gram-negative strains used as target strains, apart from three strains, namely *Lactococcus lactis* ACA-DC 46 (Formaela cheese isolate), and *E. faecium* ACA-DC 3350 and ACA-DC 3359 (both Feta cheese isolates). More precisely, the milk CFCS of *L. lactis*, as well as the MRS CFCSs of both *E. faecium* strains gave a “borderline” inhibition (*i.e.*, less colonies of the target strain around the well) towards *Porphyromonas gingivalis* DSM 20709^T^. Treatment with proteinase K revealed that this antimicrobial effect was not due to bacetriocin(s).

Regarding the Gram-positive target strains tested, only three LAB strains, namely *Lactobacillus fermentum* ACA-DC 179 (Kasseri cheese isolate), *Lactobacillus plantarum* ACA-DC 269 (Feta cheese isolate) and *Streptococcus macedonicus* ACA-DC 198 (Kasseri cheese isolate), showed antimicrobial activity towards *Streptococcus* strains (Table 2). More specifically, the MRS CFCS of *L. fermentum* ACA-DC 179 and milk CFCS of *L. plantarum* ACA-DC 269 were active against *S. oralis* LMG 14532^T^. On the other hand, the milk CFCS of *S. macedonicus* ACA-DC 198 exhibited inhibitory activity against *S. oralis* LMG 14532^T^, *S. sanguinis* DSM 20068 and *S. gordonii* LMG 14518^T^, while the M17 CFCS of *S. macedonicus* ACA-DC 198 towards *S. sanguinis* DSM 20068 and *S. gordonii* LMG 14518^T^. In all cases, the antimicrobial activity increased with the respective 10-fold concentrated CFCSs (Table 2). No activity was detected with both M17 and milk CFCS of *L. fermentum* ACA-DC 179, as well as the MRS CFCS of *S. macedonicus* ACA-DC 198 in accordance to previous experiments [13]. In addition, no activity was detected with both MRS and M17 CFCS of *L. plantarum* ACA-DC 269. In addition, the proteinaceous nature of the substances responsible for all inhibitions observed was verified after treatment with proteinase K that resulted in complete loss of antimicrobial activity.

Lactic acid bacteria, and especially strains of the genera *Lactobacillus* and *Streptococcus*, are known to play a role in the health of the oral cavity [14]. In the present study, the rarity of bacteriocins produced by food-related LAB that were active against oral LAB was unexpected. Despite the presence of at least 41 bacteriocin producers, corresponding to ≥17% of the 236 LAB strains of food origin as mentioned above, six were able to inhibit the oral pathogenic or opportunistic pathogenic bacteria tested as target strains. Among these six LAB strains, only three showed antimicrobial activity that could be correlated to bacteriocin production, representing ~1.3% of the strains tested. This difference of more than 10-fold in the number of strains inhibiting food vs. oral indicator strains is intriguing given the close phylogenetic relationship between food and oral LAB that should favor the existence of bacteriocins produced by members of the one group active against members of the other. It could be hypothesized that a correlation between the ecological niche and the antimicrobial spectrum of bacteriocins exists. Bacteriocins produced by bacteria in an ecological niche may have evolved to target mostly species/strains present in this particular niche. However, more experimental evidence is needed to validate this hypothesis.

### 2.2. Sensitivity of Oral Streptococci towards the Antimicrobial Activity of the Food LAB Strains

Since the potency of the antimicrobial substance is the most important factor, we preceded with *in vitro* inhibition assays in order to determine the effectiveness of the antimicrobials produced by food LAB. In addition, since oral bacteria have been suggested to adopt a feast and famine lifestyle in the oral cavity [15], we also attempted to assess the sensitivity of the target strains in different growth phases. Concerning the antimicrobial activity of the two *Lactobacillus* strains, only the MRS CFCS of *L. fermentum* ACA-DC 179 was included in these experiments as it was the most active one. In addition, only the milk CFCS of *S. macedonicus* ACA-DC 198 was tested as it was active towards more target strains than the M17 CFCS of the same strain. The antimicrobial activity of the two active CFCSs was studied against target cells corresponding to three different phases of bacterial growth (lag, logarithmic and stationary). In all experiments, the 10-fold concentrated supernatants of *L. fermentum* ACA-DC 179 and *S. macedonicus* ACA-DC 198 were used.

The antimicrobial activity of the MRS CFCS of *L. fermentum* against *S. oralis* LMG 14532^T^ was not evident by the *in vitro* inhibition assay. Only in the case of the stationary phase cells, a viability difference, less than 1 log, was detected between *S. oralis* cells treated with the supernatant and those treated with the control sample (data not shown).

**Figure 1:**
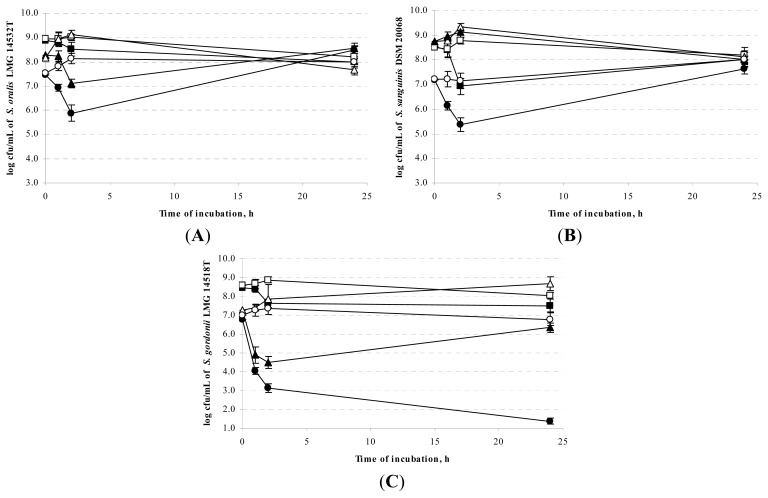
Antimicrobial activity of the 10-fold concentrated milk CFCS of *S. macedonicus* ACA-DC 198 (filled symbols) or the control sample (empty symbols; 10-fold concentrated milk containing yeast extract, pH 6.5) against stationary (squares), logarithmic (triangles), and lag phase (circles) cells of (**A**) *S. oralis* LMG 14532^T^; (**B**) *S. sanguinis* DSM 20068; and (**C**) *S. gordonii* LMG 14518^T^.

On the contrary, growth phase of the three *Streptococcus* strains affected their sensitivity to the milk CFCS of *S. macedonicus* ACA-DC 198. As shown in Figure 1, in most cases lag and logarithmic phase cells were killed rapidly (≥99% cell-death within the first 2 h). In details, for *S. oralis* LMG 14532^T^ (Figure 1A), viability differences were detected mainly for lag and logarithmic phase cells (2 log decrease after 2 h of incubation). For *S. sanguinis* DSM 20068, the milk CFCS of *S. macedonicus* affected similarly both lag and stationary phase cells (2 log decreases after 2 h of incubation) but not the logarithmic phase cells (Figure 1B). Finally, *S. gordonii* cells at lag phase were more sensitive (4 log decrease after 2 h of incubation) than logarithmic phase cells (3 log decrease after 2 h of incubation) while less activity was detected against stationary phase cells (Figure 1C). A recovery of cells after 24 h of incubation was observed that in our opinion deserves further investigation.

According to the inhibition assays performed, lag phase cells of all three sensitive *Streptococcus* strains were the most sensitive to the antimicrobial compound(s) of the milk CFCS of *S. macedonicus* ACA-DC 198. Differences in the effect of the target cells’ growth phase on their sensitivity to active bacteriocins or bacteriocin-like inhibitory substances have also been reported in the past [16–18]. Lag and exponential phase cells seem to be less resistant than stationary phase cells as during these phases biosynthesis of the target cell membrane is more vigorous, enhancing the initial electrostatic attraction of the bacteriocin that is thought to be the driving force for subsequent events [19].

### 2.3. FT-IR Analysis

**Figure 2:**
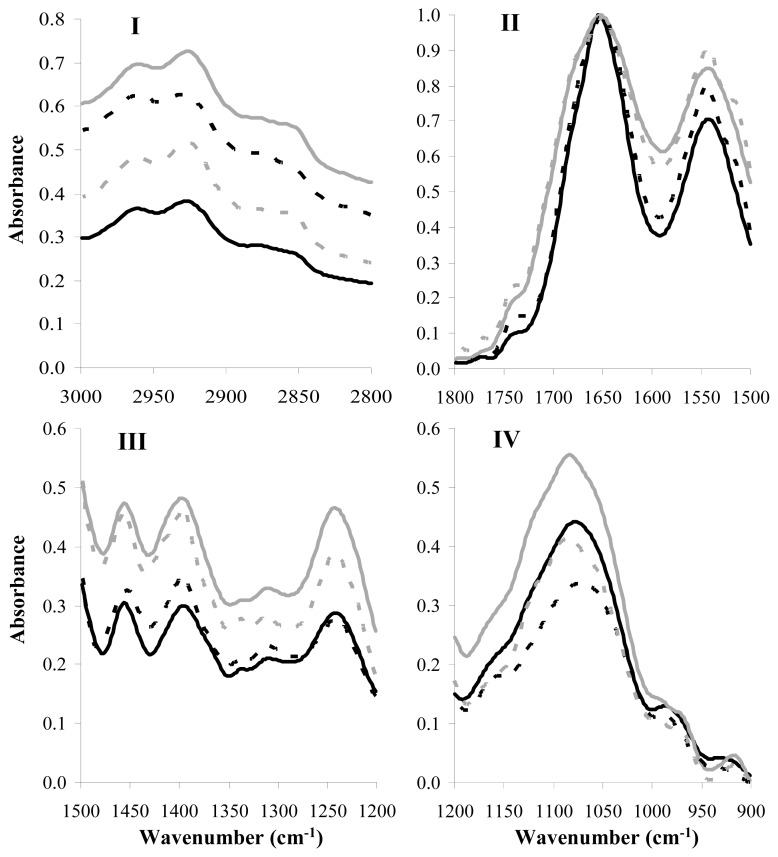
Mean FT-IR spectra of *S. gordonii* LMG 14518^T^ cells in the four characteristic spectral regions (Regions **I** to **IV**). In all spectral regions, target cells were incubated with the: (a) milk CFCS (10-fold concentrated, pH 6.5) of *S. macedonicus* ACA-DC 198 (black line); (b) control sample 1 (skim milk containing yeast extract, 10-fold concentrated, pH 6.5) (grey line); (c) pure bacteriocin sample (discontinuous black line); or (d) control sample 2 (sterile double distilled water) (discontinuous grey line). Each mean spectrum presented is the average of spectra recorded from six independent experiments.

The FT-IR spectroscopy was applied in order to specify global biochemical changes of target *Streptococcus* cells after incubation with the 10-fold concentrated milk CFCS of *S. macedonicus* ACA-DC 198. It has been previously established that in milk *S. macedonicus* produces the bacteriocin macedocin [20]. We also included samples of the target strains treated with the pure bacteriocin molecule for comparison. We adjusted the concentration of purified macedocin so as to be equal in arbitrary anits (AU)/mL to that of the CFCS during treatment. For all target strains, FT-IR spectra were recorded for lag phase cells treated for 2 h with the active supernatant or pure bacteriocin. Figure 2 shows the mean FT-IR spectra of *S. gordonii* LMG 14518^T^ cells in all four characteristic spectral regions [21,22]. Region I (3000 to 2800 cm^−1^) is the region dominated by the fatty acids of the bacterial cell membrane, and peaks at 2960 cm^−1^, 2925 cm^−1^, and 2860 cm^−1^ are assigned to the asymmetric or symmetric stretches of their methyl or methylene groups [23,24]. Region II (1800 to 1500 cm^−1^) reflects mainly the proteinaceous content of the cells as the two intense peaks at 1650 and 1550 cm^−1^ are due to the vibrations of amide I and II bands, respectively [25]. Region III (1500 to 1200 cm^−1^) is considered a mixed region, as it is informative for proteins, fatty acids, and other phosphate-carrying compounds. Peak at 1455 cm^−1^ can be assigned to the asymmetric bending of methyl or methylene groups of proteins, while peak at 1400 cm^−1^ can be attributed to the symmetric bending of the same groups as well as the C-O symmetric stretching of carbohydrates. In addition, peak at 1240 cm^−1^ is due to the symmetric stretching of phosphodiester groups in nucleic acids or phospholipids [26]. Finally, region IV (1200 to 900 cm^−1^) is the region influenced by polysaccharides of the cell wall, as the broad band at 1100 to 950 cm^−1^ arises from the stretching vibrations of the C-O polysaccharides moieties [27].

**Figure 3:**
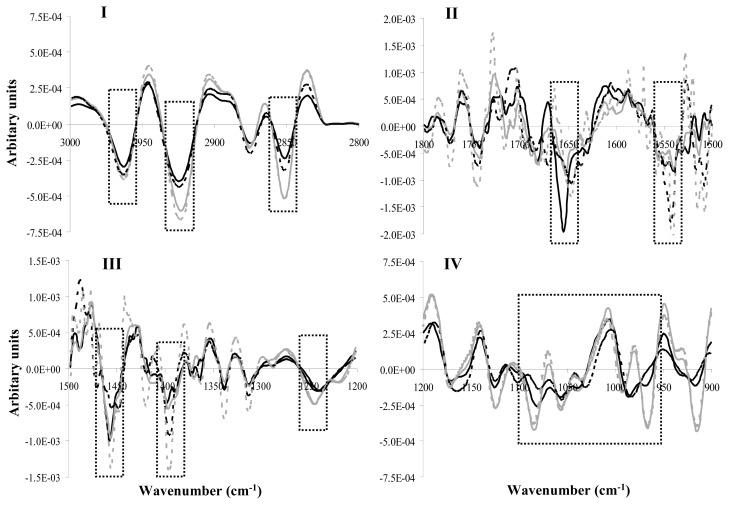
Second derivative transformation of the mean spectrum of *S. gordonii* LMG 14518^T^ cells in the four characteristic spectral regions (Regions **I** to **IV**). In all spectral regions, target cells were incubated with the: (a) milk CFCS (10-fold concentrated, pH 6.5) of *S. macedonicus* ACA-DC 198 (black line); (b) control sample 1 (skim milk containing yeast extract, 10-fold concentrated, pH 6.5) (grey line); (c) pure bacteriocin sample (discontinuous black line); or (d) control sample 2 (sterile double distilled water) (discontinuous grey line). Each mean spectrum presented is the average of spectra recorded from six independent experiments. Dotted boxes highlight important spectral regions discussed in the text.

Differences observed in all spectral regions were more discernible after second derivative transformation (Figure 3) as expected according to previous studies [28,29].

**Figure 4:**
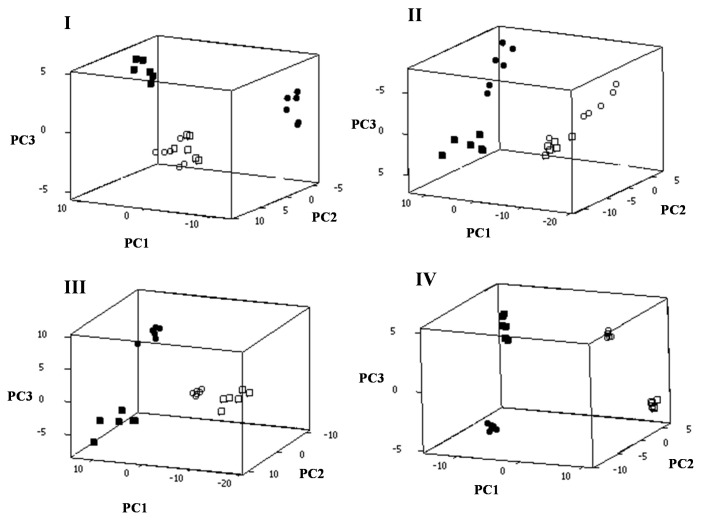
PCA of second derivative transformed FT-IR spectra of *S. gordonii* LMG 14518^T^ cells in the four characteristic spectral regions (Regions **I** to **IV**). In all spectral regions, target cells were incubated with the: (a) milk CFCS (10-fold concentrated, pH 6.5) of *S. macedonicus* ACA-DC 198 (●); (b) control sample 1 (skim milk containing yeast extract, 10-fold concentrated, pH 6.5) (○); (c) pure bacteriocin sample (■); or (d) control sample 2 (sterile double distilled water) (□). PCA was performed for the second derivative transformed spectra recorded from six independent experiments.

Principal component analysis (PCA) was performed for each spectral region separately and distinct sample clusters were observed among the cells of *S. gordonii* LMG 14518^T^ treated with the CFCS, the pure bacteriocin sample and those treated with the control samples (milk supernatant or distilled water) in all spectral regions (Figure 4). Discrete grouping of samples was readily evident within the first three PCs, accounting for 87% to 98% of the variability in the original data depending on the spectral region. These findings clearly demonstrate that the bacteriocin(s) activity within the CFCS of the *S. macedonicus* culture had resulted in alterations of the composition of all major cellular constituents of *S. gordonii*. Similar results were obtained when *S. sanguinis* DSM 20068 and *S. oralis* LMG 14532^T^ were used as target strains (data not shown).

We detected important changes in the FT-IR spectral features for the target strain *S. gordonii* LMG 14518^T^ after treatment with the CFCS or the purified macedocin. Non-treated and treated cells were clustered in distinct groups according to the PCA of their second derivative transformed FT-IR spectra. Consequently, our findings clearly support that the chemical composition of the target cells changed radically due to the antimicrobial effect of the milk CFCS of *S. macedonicus* ACA-DC 198 or the bacteriocin treatment. The magnitude of the alterations on the cellular composition of the sensitive cells, determined during FT-IR analysis, seems to be extensive (*i.e.*, affecting all major cellular constituents) and coincides with the detrimental effect of the antimicrobial compound(s). In fact, it could be hypothesized that these changes in the chemistry of the cell could be the reason for the potency of the bacteriocin produced by *S. macedonicus* ACA-DC 198. Nevertheless, the actual underlying mechanism needs to be elucidated.

It should be emphasized that control samples could be readily distinguished from the treated samples within the first PC that carries the major part of the variability within the dataset. In fact, the differences in PC values for the two control groups were significantly less than the difference of each control group with its respective treated sample (*i.e*., treated with CFCS or pure baceriocin), indicating that the chemical composition of control samples was similar. For example, the two control groups occupied the same space in the PC graph in the case of spectral region I. Furthermore, treated samples also exhibited minor differences in the PC1 axis, supporting that chemical changes at the cellular level have the same major trend with the exception of spectral region I, where the two groups of treated samples were distributed in opposite directions of the control samples. This phenomenon deserves further investigation, since it indicates that substances in the CFCS that are not present in the purified macedocin preparation may also contribute to the observed changes (and potentially the killing effect).

## 3. Experimental Section

### 3.1. Bacterial Strains and Culture Conditions

A total of 236 LAB strains, namely 139 lactobacilli and 97 cocci, were included in the present study as potential producers of antimicrobial activity (Table 1). Identification of LAB strains was performed with 16s rRNA gene sequencing followed by phenotypic tests, including API 50 CH tests (bioMérieux, Inc., Marcy l’Etoile, France). The strains were isolated mainly from dairy products and are held at the ACA-DC Collection (Laboratory of Dairy Research, Agricultural University of Athens, Athens, Greece). They were stored at −80 °C in MRS (Biokar Diagnostics, Beauvais, France) or M17 broth (Biokar Diagnostics), supplemented with 20% (*v*/*v*) glycerol.

Ten strains, among them 3 Gram-negative and 7 Gram-positive, belonging to bacteria species related to the oral health were used as target strains. *Fusobacterium nucleatum* subsp. *vincentii* DSM 19507^T^ and *Porphyromonas gingivalis* DSM 20709^T^ were grown in BHI supplemented with yeast extract (0.5% *w*/*v*, Biokar Diagnostics), hemin (5 μg/mL, Sigma, St. Louis, MO, USA) and menadione (1 μg/mL, Sigma) at 37 °C, anaerobically (GasPak kit, Beckton Dickinson, USA). *Aggregatibacter actinomycetemcomitans* DSM 11123 was cultured in BHI (Biokar Diagnostics) enriched with yeast extract (0.5% *w*/*v*, Biokar Diagnostics) at 37 °C. Finally, *Staphylococcus aureus* DSM 21705 and six *Streptococcus* strains, namely *Streptococcus gordonii* LMG 14518^T^, *Streptococcus mutans* LMG 14558^T^, *Streptococcus oralis* LMG 14532^T^, *Streptococcus salivarius* LMG 11489^T^, *Streptococcus sanguinis* DSM 20068, and *Streptococcus sobrinus* LMG 14641^T^, were grown in BHI (Biokar Diagnostics) at 37 °C.

The target strains presented in supplementary Table 1 were cultivated as follows: *Clostridium tyrobutyricum* NCDO 1754 was grown anaerobically (GasPak kit) in RCM (Biokar Diagnostics) at 37 °C, *Listeria innocua* BL86/26 in BHI at 30 °C, lactobacilli in MRS at appropriate temperatures and enterococci, lactococci and streptococci in M17, also at appropriate temperatures.

### 3.2. Preparation of Cell-Free Culture Supernatants (CFCSs) and of Pure Macedocin

CFCSs of the potential producer strains were collected after growth in skim milk (10% *w*/*v*, Oxoid) containing 0.3% (*w*/*v*) yeast extract (Biokar Diagnostics), MRS and M17 broth. Fresh overnight culture supernatants were collected by centrifugation (12,000 ×*g*, 15 min, 4 °C), adjusted to pH 6.5 and filtered (0.22 μm Millex Syringe Filter, Millipore, Billerica, MA, USA). Skim milk containing yeast extract, MRS and M17 broth also adjusted to pH 6.5 and filtered were used as controls. Neutralizing the pH of the supernatant was considered necessary to avoid false positive results in the WDA due to acidity-mediated inhibition of the indicator strain.

In order to obtain the 10-fold concentrated CFCSs, pellets of the above supernatants, obtained after ammonium sulfate precipitation (50% saturation), were diluted in the appropriate volume of sterilized distilled water. Non-inoculated growth medium (skim milk containing yeast extract, MRS and M17), adjusted to pH 6.5, filtered and concentrated (10-fold) after ammonium sulfate treatment were used as controls. Before use, all concentrated supernatants were once more centrifuged (12,000 ×*g*, 15 min, 4 °C) in order to avoid further precipitation during the experiments.

CFCSs of strains presented in supplementary Table 1 were prepared as above from fresh overnight cultures of lactobacilli, leuconostocs and pediococci grown in MRS and enterococci, lactococci and streptococci grown in M17, with the exception of *Streptococcus macecdonicus* ACA-DC 198 that was grown in skim milk supplemented with 0.3% (*w*/*v*) yeast extract.

Pure macedocin was prepared as previously described [20]. After dialysis, active fractions were lyophilized and resuspended in distilled water (data not shown).

### 3.3. Detection of Antimicrobial Activity

CFCSs were screened for antimicrobial activity using the well diffusion assay (WDA). Briefly, 5 × 10^5^–1 × 10^6^ cfu/mL of the target strain was incorporated into the appropriate soft agar (1% *w*/*v*) plates. CFCSs (50 μL) of the potential producer strains were transferred to holes (5 mm diameter) drilled into the agar. The plates were then incubated at the appropriate conditions, depending on the target strain, and the antimicrobial activity was recorded as growth-free inhibition zones (diameter) around the well. To assess the effect of proteinase K on the CFCSs antimicrobial activity, proteinase K (Applichem, Darmstadt, Germany) was added in a well next to the well containing the CFCSs at a final concentration of 2 mg/mL. The effect was attributed to the production of bacteriocins if the inhibition zone totally disappeared after proteinase K treatment.

### 3.4. Inhibition Assays

The inhibitory activity of CFCSs towards sensitive strains was studied by an *in vitro* inhibition assay designed to target cells at different bacterial growth phases, namely lag, logarithmic and stationary phase cells.

To obtain target cells, an inoculum (2% *v*/*v*) of fresh 18 h culture was transferred to 5 mL of BHI broth and incubated at 37 °C. For lag phase cells, cells were incubated for 2 h (*S. oralis* LMG 14532^T^ and *S. sanguinis* DSM 20068) or 2.5 h (*S. gordonii* LMG 14518^T^). For logarithmic phase cells, cells were incubated for 3, 4 and 5 h (*S. oralis* LMG 14532^T^, *S. sanguinis* DSM 20068, and *S. gordonii* LMG 14518^T^, respectively). Finally, for stationary phase cells, cells were incubated for 18 h for all three *Streptococcus* strains. After incubation, cells were collected by centrifugation, washed twice with phosphate-buffered saline (PBS; pH 7.2) and resuspended in the same volume of fresh BHI broth (5 mL). Target cell suspensions were then mixed with equal volume (5 mL) of active CFCS or the control supernatant and incubated at 37 °C for 24 h. At various time intervals, the surviving cells were determined by plating on the appropriate growth medium. Three independent experiments were performed.

### 3.5. FT-IR Sample Preparation and Measurements

Lag phase cell suspensions of the target strain (5 mL) were mixed with equal volume (5 mL) of active CFCS or the control supernatant and incubated at 37 °C. Purified macedocin was used in the same concentration to that of the CFCS in AU/mL. After 2 h of incubation, cells were collected by centrifugation, washed twice with PBS (pH 7.2) and resuspended in 30 μL of sterile double-distilled water. FT-IR sample preparation and measurements were performed according to the methodology described before [28]. In brief, 10 μL of the resulting suspension were spotted at the center of an IR transparent ZnSe optical disc. Samples were then air dried under laminar flow in order to achieve the formation of a homogenous dried film of bacterial cells. Nicolet 6700 spectrometer (DTGS detector; Nichrome source; KBr beamsplitter; Thermo Electron Corporation, Madison, WI, USA) was used to record FT-IR bacterial spectra. Spectra were recorded in absorbance between wave numbers 4000 to 600 cm^−1^ at a 4 cm^−1^ spectral resolution and each spectrum was obtained by co-adding 100 scans.

### 3.6. FT-IR Data Analysis

Omnic software (Version 7.3, Thermo Electron Corporation) was used to conduct spectral processing. FT-IR spectra were smoothed, baseline corrected and normalized using the automatic functions and the algorithms built into the aforementioned software. Furthermore, the second derivative transformation of each pre-processed spectrum was calculated with the Savitzky-Golay derivative filter and default parameters (7-point, 4 cm^−1^ resolution, polynomial degree 3). Data from the second derivative transformed spectra were imported into Statgraphics Centurion software (Version XV, Statpoint Technologies Inc., VA, USA) and principal component analysis (PCA) was employed in order to statistically evaluate these data.

## 4. Conclusions

After a screening of an important number of LAB food isolates for the production of bacteriocins active against a set of known bacteria implicated in oral disease, we report that this seems to be a rare property. Overall, only three out of 236 LAB strains, namely *L. plantarum* ACA-DC 269, *L. fermentum* ACA-DC 179 and *S. macedonicus* ACA-DC 198 showed inhibitory activity against bacteria related to oral health that could be attributed to bacteriocin(s) present in their cell-free culture supernatants, even though at least 41 bacteriocin-producing LAB strains were included in the set of strains used in this experiment. This conclusion is rather unexpected due to the close phylogenetic relationship between food- and oral-related LAB. The effect of the *S. macedonicus* ACA-DC 198 milk CFCS or purified macedocin was more prominent against target cells at the lag phase of growth and could be correlated to major alterations in the chemistry of the cells as a whole. Thus, the antimicrobial compounds investigated here could be further studied for their role in oral infections. Our findings indicate that this chase for appropriate antibiotics towards oral pathogens within food LAB must be continued.

## Supplementary Information



## Figures and Tables

**Table 1:** Genus and species of the lactic acid bacteria used as potential producers of bacteriocins.

Genus/species	Number of strains
***Bacilli***	

*Lactobacillus acidipiscis*	1
*Lactobacillus acidophilus*	3
*Lactobacillus brevis*	2
*Lactobacillus delbrueckii* subsp. *bulgaricus*	11
*Lactobacillus delbrueckii* subsp. *delbrueckii*	1
*Lactobacillus delbrueckii* subsp. *lactis*	4
*Lactobacillus fermentum*	3
*Lactobacillus gasseri*	6
*Lactobacillus paracasei* subsp. *paracasei*	17
*Lactobacillus paracasei* subsp. *tolerans*	1
*Lactobacillus plantarum*	52
*Lactobacillus rennini*	11
*Lactobacillus rhamnosus*	1
*Lactobacillus sanfransinscensis*	1
*Lactobacillus* sp.	25

***Cocci***	

*Enterococcus faecalis*	3
*Enterococcus faecium*	2
*Lactococcus lactis*	41
*Leuconostoc lactis*	2
*Leuconostoc mesenteroides* subsp. *dextranicum*	4
*Pediococcus pentosaceus*	7
*Streptococcus bovis*	1
*Streptococcus macedonicus*	5
*Streptococcus thermophilus*	32

**Table 2:** Antimicrobial activity of cell-free culture supernatants (CFCSs) of *L. fermentum* ACA-DC 179, *L. plantarum* ACA-DC 269 and *S. macedonicus* ACA-DC 198 towards three *Streptococcus* strains as determined by the well diffusion assay.

Target strain	Inhibition (diameter; mm ± SD) a							

	*L. fermentum* ACA-DC 179		*L. plantarum* ACA-DC 269		*S. macedonicus* ACA-DC 198			

	MRS CFCS	10-fold concentrated MRS CFCS	Milk CFCS	10-fold concentrated milk CFCS	Milk CFCS	10-fold concentrated milk CFCS	M17 CFCS	10-fold concentrated M17 CFCS
*S. oralis* LMG 14532^T^	0.7 ± 0.10	1.1 ± 0.10	0.5 ± 0.06	0.9 ± 0.06	1.0 ± 0.06	1.1 ± 0.10	– b	–
*S. sanguinis* DSM 20068	–	–	–	–	1.0 ± 0.06	1.1 ± 0.12	0.9 ± 0.06	1.2 ± 0.12
*S. gordonii* LMG 14518^T^	–	–	–	–	1.2 ± 0.06	1.6 ± 0.10	1.0 ± 0.06	1.4 ± 0.12

a Values are means of the results of three independent experiments;

b No inhibition.

## References

[1] (2002). Joint FAO/WHO, Report of a Joint FAO/WHO expert consultation on guidelines for the evaluation of probiotics in food.

[2] Bosch M., Nart J., Audivert S., Bonachera M.A., Alemany A.S., Fuentes M.C., Cune J. (2012). Isolation and characterization of probiotic strains for improving oral health. Arch. Oral. Biol.

[3] Snel J., Marco M.L., Kingma F., Noordman W.M., Rademaker J., Kleerebezem M. (2011). Competitive selection of lactic acid bacteria that persist in the human oral cavity. Appl. Environ. Microbiol.

[4] Kolenbrander P.E., Palmer R.J., Rickard A.H., Jakubovics N.S., Chalmers N.I., Diaz P.I. (2006). Bacterial interactions and successions during plaque development. Periodontol. 2000.

[5] Kang M.S., Oh J.S., Lee H.C., Lim H.S., Lee S.W., Yang K.H., Choi N.K., Kim S.M. (2011). Inhibitory effect of *Lactobacillus reuteri* on periodontopathic and cariogenic bacteria. J. Microbiol.

[6] Bonifait L., Chandad F., Grenier D. (2009). Probiotics for oral health: Myth or reality?. J. Can. Dent. Assoc.

[7] Stamatova I., Meurman J.H. (2009). Probiotics and periodontal disease. Periodontol. 2000.

[8] Li X., Kolltveit K.M., Tronstad L., Olsen I. (2000). Systemic diseases caused by oral infection. Clin. Microbiol. Rev.

[9] Nakano K., Inaba H., Nomura R., Nemoto H., Takeda M., Yoshioka H., Matsue H., Takahashi T., Taniguchi K., Amano A. (2006). Detection of cariogenic Streptococcus mutans in extirpated heart valve and atheromatous plaque specimens. J. Clin. Microbiol.

[10] Takarada K., Kimizuka R., Takahashi N., Honma K., Okuda K., Kato T. (2004). A comparison of the antibacterial efficacies of essential oils against oral pathogens. Oral Microbiol. Immunol.

[11] Kirkup B.C. (2006). Bacteriocins as oral and gastrointestinal antibiotics: Theoretical considerations, applied research, and practical applications. Curr. Med. Chem..

[12] Wescombe P.A., Heng N.C., Burton J.P., Chilcott C.N., Tagg J.R. (2009). Streptococcal bacteriocins and the case for Streptococcus salivarius as model oral probiotics. Future Microbiol.

[13] Zoumpopoulou G., Foligne B., Christodoulou K., Grangette C., Pot B., Tsakalidou E. (2008). *Lactobacillus fermentum* ACA-DC 179 displays probiotic potential *in vitro* and protects against trinitrobenzene sulfonic acid (TNBS)-induced colitis and Salmonella infection in murine models. Int. J. Food Microbiol.

[14] Meurman J.H., Stamatova I, Lahtinen S., Ouwehand A.C., Salminen S., von Wright A. (2012). Lactic acid bacteria in oral health. Lactic Acid Bacteria: Microbiological and Functional Aspects.

[15] Lemos J.A., Abranches J., Burne R.A. (2005). Responses of cariogenic streptococci to environmental stresses. Curr. Issues Mol. Biol.

[16] Cao-Hoang L., Marechal P.A., Le-Thanh M., Gervais P. (2008). Synergistic action of rapid chilling and nisin on the inactivation of Escherichia coli. Appl. Microbiol. Biotechnol.

[17] Dykes G.A., Moorhead S.M. (2002). Combined antimicrobial effect of nisin and a listeriophage against *Listeria monocytogenes* in broth but not in buffer or on raw beef. Int. J. Food Microbiol.

[18] Schobitz R., Suazo V., Costa M., Ciampi L. (2003). Effects of a bacteriocin-like inhibitory substance from *Carnobacterium piscicola* against human and salmon isolates of *Listeria monocytogenes*. Int. J. Food Microbiol.

[19] Deegan L.H., Cotter P.D., Hill C., Ross P. (2006). Bacteriocins: Biological tools for bio-preservation and shelf-life extension. Int. Dairy Fed.

[20] Georgalaki M.D., van den Berghe E., Kritikos D., Devreese B., van Beeumen J., Kalantzopoulos G., de Vuyst L., Tsakalidou E. (2002). Macedocin, a food-grade lantibiotic produced by *Streptococcus macedonicus* ACA-DC 198. Appl. Environ. Microbiol.

[21] Alvarez-Ordonez A., Mouwen D.J., Lopez M., Prieto M. (2011). Fourier transform infrared spectroscopy as a tool to characterize molecular composition and stress response in foodborne pathogenic bacteria. J. Microbiol. Methods.

[22] Lu X., Al-Qadiri H., Lin M., Rasco B. (2011). Application of mid-infrared and raman spectroscopy to the study of bacteria. Food Bioprocess Technol.

[23] Al-Qadiri H.M., Lin M., Cavinato A.G., Rasco B.A. (2006). Fourier transform infrared spectroscopy, detection and identification of *Escherichia coli* O157:H7 and *Alicyclobacillus* strains in apple juice. Int. J. Food Microbiol.

[24] Schmitt J., Flemming H.-C. (1998). FTIR-spectroscopy in microbial and material analysis. Int. Biodeterior. Biodegrad.

[25] Kansiz M., Heraud P., Wood B., Burden F., Beardall J., McNaughton D. (1999). Fourier transform infrared microspectroscopy and chemometrics as a tool for the discrimination of cyanobacterial strains. Phytochemistry.

[26] Zeroual W., Choisy C., Doglia S.M., Bobichon H., Angiboust J.F., Manfait M. (1994). Monitoring of bacterial growth and structural analysis as probed by FT-IR spectroscopy. Biochim. Biophys. Acta.

[27] Filip Z., Hermann S. (2001). An attempt to differentiate *Pseudomonas* spp. and other soil bacteria by FT-IR spectroscopy. Eur. J. Soil Biol.

[28] Papadimitriou K., Boutou E., Zoumpopoulou G., Tarantilis P.A., Polissiou M., Vorgias C.E., Tsakalidou E. (2008). RNA arbitrarily primed PCR and fourier transform infrared spectroscopy reveal plasticity in the acid tolerance response of Streptococcus macedonicus. Appl. Environ. Microbiol.

[29] Zoumpopoulou G., Papadimitriou K., Polissiou M.G., Tarantilis P.A., Tsakalidou E. (2010). Detection of changes in the cellular composition of *Salmonella* enterica serovar Typhimurium in the presence of antimicrobial compound(s) of *Lactobacillus* strains using Fourier transform infrared spectroscopy. Int. J. Food Microbiol.

