# Supplementary material for: Incidence of Bacteriocins Produced by Food-Related Lactic Acid Bacteria Active towards Oral Pathogens
Source: Int J Mol Sci. 2013 Feb 26;14(3):4640–54. doi: 10.3390/ijms14034640 (PMC3634443; doi:10.3390/ijms14034640)
Supplement: Supplementary file 1 [file ijms-14-04640-s001.pdf]

# Supporting Information

**Table S1.** Strains within the set of 236 strains of the lactic acid bacteria screened in this study known to produce bacteriocins against food-related indicator strains.

| No. | Producer strain | Source | Indicator strains | | | | | | | | | | |
|---|---|---|---|---|---|---|---|---|---|---|---|---|---|
| | | | Bacilli | | | | | | Cocci | | | | |
| | | | *Clostidium tyrobutyricum NCDO 1754* | *Lactobacillus delbrueckii* subsp. *bulgaricus ACA-DC 84* | *Lactobacillus helveticus ATCC 15009* | *Lactobacillus plantarum ACA-DC 125* | *Lactobacillus sakei LMG 2313* | *Listeria innocua BL86/26* | *Enterococcus faecalis ACA-DC 138* | *Enterococcus faecalis EF-1* | *Lactococcus lactis ACA-DC 49* | *Lactococcus lactis CNRZ 117* | *Streptococcus thermophilus ACA-DC 4* |
| | **Bacilli** | | | | | | | | | | | | |
| 1 | *Lactobacillus acidophilus* ACA-DC 4002 | Sourdough | | | | | | √ | | | √ | √ | |
| 2 | *Lactobacillus fermentum* ACA-DC 179 | Kasseri cheese | | | | | | | | | | | √ |
| 3 | *Lactobacillus delbrueckii subsp. bulgaricus* ACA-DC 321 | Staka chesse | | | | | | | √ | √ | | | |
| 4 | *Lactobacillus paracasei* ACA-DC 116 | Feta brine | | | | | | √ | | | √ | √ | |
| 5 | *Lactobacillus paracasei* ACA-DC 3353 | Kasseri cheese | √ | | | √ | | | | | | | |
| 6 | *Lactobacillus plantarum* ACA-DC 146 | Feta brine | | √ | | | | √ | | | √ | | |
| 7 | *Lactobacillus plantarum* ACA-DC 147 | Feta brine | | | | | | √ | | | √ | √ | |
| 8 | *Lactobacillus plantarum* ACA-DC 201 | Kasseri cheese | | | | √ | √ | | | | | | |
| 9 | *Lactobacillus plantarum* ACA-DC 279 | Xynotyri cheese | | | | | | √ | | | √ | √ | √ |
| 10 | *Lactobacillus plantarum* ACA-DC 280 | Xynotyri cheese | | √ | | | | √ | | | √ | | |
| 11 | *Lactobacillus plantarum* ACA-DC 281 | Xynotyri cheese | | | | | | √ | | | | √ | √ |
| 12 | *Lactobacillus plantarum* ACA-DC 288 | Xynotyri cheese | | | | √ | √ | | | | √ | | |
| 13 | *Lactobacillus plantarum* ACA-DC 424 | Arseniko cheese | | | √ | | √ | | | | | | |
| 14 | *Lactobacillus plantarum* ACA-DC 490 | Arseniko cheese | | | | | | √ | | | | √ | |
| 15 | *Lactobacillus plantarum* ACA-DC 2293 | Feta cheese | | √ | | √ | √ | | | | √ | | |
| 16 | *Lactobacillus plantarum* ACA-DC 2350 | Feta cheese | | | | | √ | √ | | | | | √ |
| 17 | *Lactobacillus plantarum* ACA-DC 2414 | Feta cheese | | | | | √ | √ | | | √ | | |
| 18 | *Lactobacillus plantarum* ACA-DC 2580 | Feta cheese | | √ | | √ | | | | | | | |
| 19 | *Lactobacillus plantarum* ACA-DC 2640 | Feta cheese | | | | | √ | √ | | | √ | | |
| 20 | *Lactobacillus plantarum* ACA-DC 2652 | Feta cheese | | | | √ | √ | √ | | | | | |
| 21 | *Lactobacillus plantarum* ACA-DC 2830 | Feta cheese | | | | | | √ | | | √ | √ | |
| 22 | *Lactobacillus plantarum* ACA-DC 2881 | Feta cheese | | | √ | | √ | √ | | | | | |

**Table S1.** *Cont.*

| No. | Producer strain | Source | Indicator strains | | | | | | | | | | |
|---|---|---|---|---|---|---|---|---|---|---|---|---|---|
| | | | Bacilli | | | | | | Cocci | | | | |
| | | | ***Clostidium tyrobutyricum* NCDO 1754** | ***Lactobacillus delbrueckii* subsp. *bulgaricus* ACA-DC 84** | ***Lactobacillus helveticus* ATCC 15009** | ***Lactobacillus plantarum* ACA-DC 125** | ***Lactobacillus sakei* LMG 2313** | ***Listeria innocua* BL86/26** | ***Enterococcus faecalis* ACA-DC 138** | ***Enterococcus faecalis* EF-1** | ***Lactococcus lactis* ACA-DC 49** | ***Lactococcus lactis* CNRZ 117** | ***Streptococcus thermophilus* ACA-DC 4** |
| | **Cocci** | | | | | | | | | | | | |
| 23 | *Enterococcus faecalis* ACA-DC 3307 | Raw milk cheese | √ | | | | | | | | √ | | |
| 24 | *Enterococcus faecium* ACA-DC 434 | Arseniko cheese | | | | √ | √ | | | | | | |
| 25 | *Lactococcus lactis* ACA-DC 58 | Feta cheese | | | | | | | | √ | | | √ |
| 26 | *Lactococcus lactis* ACA-DC 59 | Feta curd | | | | | | | √ | √ | | | |
| 27 | *Lactococcus lactis* ACA-DC 60 | Kefalotyri cheese | | | | | | | √ | √ | | √ | |
| 28 | *Lactococcus lactis* ACA-DC 154 | Kasseri cheese | | √ | | | | | √ | √ | | | |
| 29 | *Lactococcus lactis* ACA-DC 4019 | Feta cheese | √ | | | | | | | | √ | √ | √ |
| 30 | *Lactococcus lactis* ACA-DC 4030 | Feta cheese | √ | √ | | | | | | | | | |
| 31 | *Lactococcus lactis* ACA-DC 4033 | Feta cheese | | | | | | | | | √ | √ | |
| 32 | *Lactococcus lactis* ACA-DC 4034 | Kasseri cheese | √ | | | | | | √ | | √ | | |
| 33 | *Leuconostoc mesenteroides* subsp.*dextranicum* ACA-DC 493 | Arseniko cheese | | | | | √ | | | | √ | √ | |
| 34 | *Pediococcus pentosaceus* ACA-DC 137 | Feta brine | | | | | √ | √ | | | | | |
| 35 | *Streptococcus macedonicus* ACA-DC 198 | Kasseri cheese | √ | √ | √ | √ | √ | | | | | √ | √ |
| 36 | *Streptococcus thermophilus* ACA-DC 7 | Yogurt | | | | | | | | | √ | √ | |
| 37 | *Streptococcus thermophilus* ACA-DC 10 | Dairy industrial mixture | | | | | | | | | √ | √ | |
| 38 | *Streptococcus thermophilus* ACA-DC 26 | Yogurt | | | | | | √ | | | | √ | |
| 39 | *Streptococcus thermophilus* ACA-DC 40 | Xynotyri cheese | | | | | | | √ | √ | √ | | |
| 40 | *Streptococcus thermophilus* ACA-DC 1691 | Feta cheese | | | | | | | √ | √ | | √ | |
| 41 | *Streptococcus thermophilus* ACA-DC 2048 | Feta cheese | | | | | | | | | √ | √ | √ |

© 2013 by the authors; licensee MDPI, Basel, Switzerland. This article is an open access article distributed under the terms and conditions of the Creative Commons Attribution license (http://creativecommons.org/licenses/by/3.0/).
